# Immunogenicity and protective activity of mRNA vaccine candidates against yellow fever virus in animal models

**DOI:** 10.1038/s41541-023-00629-7

**Published:** 2023-03-04

**Authors:** Lex G. Medina-Magües, Janine Mühe, Edith Jasny, Emily S. Medina-Magües, Nicole Roth, Jaime Lopera-Madrid, Cristhian Salas-Quinchucua, Cole Knuese, Benjamin Petsch, Jorge E. Osorio

**Affiliations:** 1 Department of Pathobiological Sciences, School of Veterinary Medicine, University of Wisconsin, Madison, WI 53706 USA; 2 Infectious Diseases Research Unit, CureVac SE, Friedrich-Miescher-Straße 15, 72076 Tübingen, Germany

**Keywords:** RNA vaccines, Vaccines, Preclinical research

## Abstract

Despite the success of the widely used attenuated yellow fever (YF) vaccine, its global supply remains a substantial barrier to implementing vaccination campaigns in endemic regions and combating emerging epidemics. In A129 mice and rhesus macaques, we evaluated the immunogenicity and protective activity of messenger RNA (mRNA) vaccine candidates encapsulated in lipid nanoparticles, expressing the pre-membrane and envelope proteins or the non-structural protein 1 of YF virus. Vaccine constructs induced humoral and cell-mediated immune responses in mice, resulting in protection against lethal YF virus infection after passive administration of serum or splenocytes from vaccinated mice. Vaccination of macaques induced sustained high humoral and cellular immune responses for at least 5 months after the second dose. Our data demonstrate that these mRNA vaccine candidates can be considered an attractive addition to the licensed YF vaccine supply based on the induction of functional antibodies correlating with protection and T-cell responses; they could alleviate the limited supply of current YF vaccines, mitigating future YF epidemics.

## Introduction

Yellow fever (YF), an acute viral hemorrhagic disease, was one of the most feared and lethal diseases for centuries^1^. The disease is endemic to Central and South America as well as sub-Saharan Africa^2^, with 200,000 cases and up to 50,000 deaths recorded every year^3,4^. YF is caused by the YF virus (YFV) of the genus *Flavivirus*, which is transmitted to humans by *Aedes aegypti* mosquitos. One strategy to limit YF outbreaks involves controlling the mosquito vector population. However, YFV is maintained in a cycle between *Aedes, Haemagogus*, or *Sabethes spp*. mosquitoes and non-human primate (NHP) reservoirs in the rainforest making complete eradication of YFV unattainable^5^.

Immunization campaigns provide the leading strategy for limiting YF outbreaks in humans. Over 80 years ago, a live-attenuated virus vaccine (YF-17D) was developed following serial passage of the virulent YFV Asibi strain. The current vaccine strains, YF 17DD (passage 195) and 17D-204 (passage 204)^6,7^, are considered safe and effective, and provide lifelong immunity after a single immunization^8,9^. Severe adverse events are rare, but severe hypersensitivity or anaphylactic reactions, YF vaccine-associated neurologic disease, and YF vaccine-associated viscerotropic disease can occur^10^. However, the Vaccine Adverse Event Reporting System reports only 3589 total events after YF immunization in the USA since 1990 (as of October 21, 2022)^11^. As a live-attenuated virus, the vaccine poses additional risks for pregnant women, infants (<6 months), seniors (>60 years old), immunocompromised individuals, and people with hypersensitivity to vaccine components, e.g., egg allergies, and is therefore contraindicated for these populations^12^. Still, vaccination is the only protection from YF since no antiviral therapies are currently available.

However, YF vaccines are scarce. Although YF vaccine production capacity was increased from 20 million to a range between 116 and 159 million doses in 2021^13^, current estimates suggest that 1.38 billion doses are needed to meet the global demand over this decade to eliminate the risk of future YF epidemics^14^. This exceeds the doses administered in the previous 80 years combined^15^. YF vaccines are produced in specific pathogen-free embryonated chicken eggs from fully characterized master seed lots. Thus, production of the vaccine is slow, laborious, and highly dependent on the availability of suitable chicken eggs. New vaccine technologies using scalable production platforms could overcome the vaccine shortages, especially in urgent need situations such as an endemic outbreak. mRNA vaccines combine fast development and manufacturing, with the ability to induce humoral and cellular immune responses and appear to have an acceptable safety profile^16^. This platform was used successfully to fight the coronavirus disease 2019 (COVID-19) pandemic, proving the superiority in manufacturing times, while being highly efficacious and showing an acceptable safety profile^17,18^.

Here, we evaluated the immunogenicity and protective activity of two mRNA-based YF vaccine candidates formulated within a lipid nanoparticle (LNP). One candidate encoded the YFV pre-membrane and envelope proteins (prM-E), responsible for virus entry into the host cell and the primary targets for neutralizing antibodies (nAbs). The other encoded the non-structural protein 1 (NS1), which is critical for viral replication and involved in immune evasion and pathogenesis^19^. Previously, vaccination with NS1 provided protection from a lethal challenge in NHPs^20^. Immunogenicity of both mRNA YF vaccine candidates was evaluated in A129 mice and rhesus macaques. The protective roles of humoral and cellular immune responses were determined by passive transfer of murine or NHP serum, or murine splenocytes into susceptible mice followed by YFV challenge. Overall, the mRNA vaccine candidates demonstrated the induction of innate immune responses as well as humoral and cellular immunity comparable to the licensed vaccine during an observation period of up to 6 months.

## Results

### YF mRNA-LNP vaccines are immunogenic and provide protection from wild-type YFV challenge in A129 mice

The vaccine’s ability to protect from YF was tested in A129 mice deficient in the interferon-alpha/beta (IFN-α/β) receptor (IFNAR^−/−^). A129 mice are susceptible to wild-type YFV (wtYFV) infection and develop a viscerotropic disease similar to human YF^21,22^, but survive inoculation with YF-17D. Seven- to eight-week-old A129 mice were vaccinated with the YF prM-E or NS1 mRNA-LNP vaccine candidates. Animals were injected intramuscularly (IM) twice 21 days apart with 0.5 µg prM-E mRNA-LNP vaccine, 0.5 µg each of the prM-E and NS1 mRNA-LNP vaccines (bivalent approach), 4 µg of the NS1 mRNA-LNP vaccine, or sodium chloride (NaCl) buffer as a negative control (placebo). One group received 2.5 µg each of the prM-E and NS1 mRNA-LNP vaccines as a single bivalent dose. The positive control group received a single injection with one-tenth of a human dose of YFV 17D-204 (i.e., Stamaril^®^) (Supplementary Fig. 1a).

**Fig. 1:**
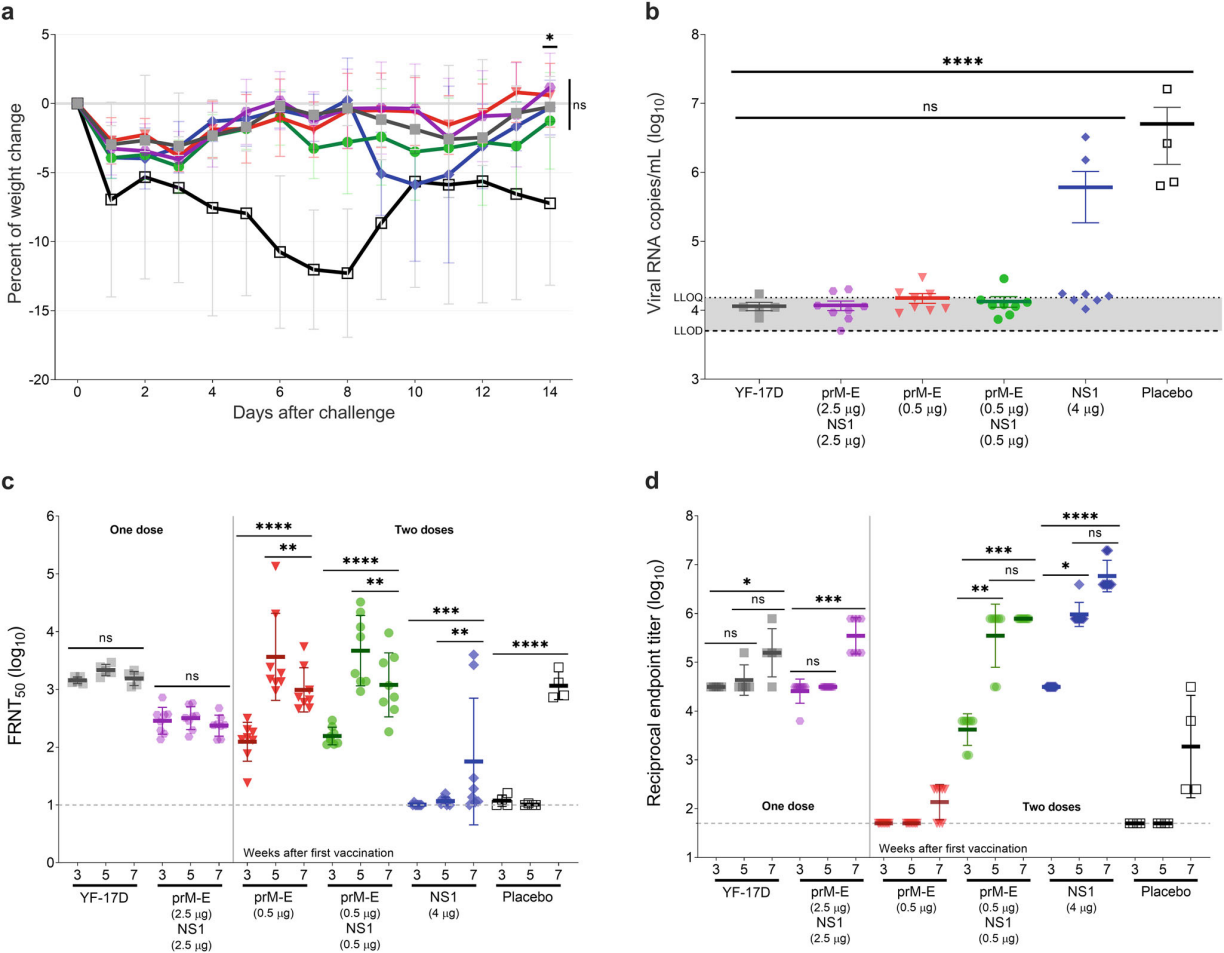
YFV-based mRNA-LNP vaccines elicit protective titers and protect against intramuscular (IM) YFV challenge in A129 mice. Mice received one immunization with one-tenth of the human dose of YF-17D vaccine (close square; gray) or bivalent YF prM-E/NS1 mRNA-LNPs (2.5 µg each) (hexagon; violet), or two immunizations with 0.5 µg of YF prM-E mRNA-LNP (inverted triangle; red), 0.5 µg each of YF prM-E and NS1 mRNA-LNPs (circle; green), 4 µg of YF NS1 mRNA-LNP (diamond; blue), or diluent as placebo control (open square; black) 21 days apart. Five weeks later, on day 35, mice were infected IM with 1 × 10^4^ plaque-forming units (PFU) of YFV BeH 622205 strain. Panel **a** shows the percentage of the body weight change (±SD) of the animals over a period of 14 days post challenge. **b** Serum viral load (line indicates GMT ± standard error of the mean [SEM]) measured 6 days after challenge by qRT-PCR. Antibody responses were measured in serum 21 days after the first vaccination (week 3), 14 days after the second vaccination (week 5), and 14 days after challenge (week 7). Panel **c** shows the neutralizing antibody titers measured by FRNT_50_ (GMT ± geometric standard deviation [GSD]). **d** Enzyme-linked immunoassay (ELISA) data showing anti-YFV NS1-specific antibody titers (GMT ± GSD). Dashed line indicates the lower limit of detection (LLOD), and dotted line shows the lower limit of quantification (LLOQ), *p < 0.05, **p < 0.01, ***p < 0.001, ****p < 0.0001, ns = not significant. One-way analysis of variance (ANOVA) followed by Dunnett’s or Dunn’s multiple comparisons test for viral load and ELISA data, respectively, or two-way ANOVA followed by Tukey’s multiple comparisons test for neutralizing antibody titers and weight changes was performed using GraphPad Prism. Statistical analysis of the FRNT_50_ titers was performed after log-transformation of the data and assessed for normality using a Q–Q (quantile-quantile) plot.

Two weeks after the second immunization, all mice were challenged with YFV BeH 622205 strain and observed for 2 weeks. All vaccinated and placebo control mice survived the challenge. Most mouse models for YF produce signs of disease only in the first weeks of life. This age-dependent resistance is probably due to the maturation of the immune system, and it is highly affected by the inoculation site^23^. Adult mice were chosen for this vaccination study to achieve optimal immune responses against the viral antigens. However, age might have affected susceptibility to the YFV challenge. Still, we did observe clinical signs of YF such as lethargy and body weight loss in the placebo group. Notably, the body weight loss in the placebo group was more pronounced and prolonged than in the YF mRNA-LNP or YF-17D vaccinated groups (Fig. 1a). For mRNA-vaccinated mice, the body weight showed a decrease 1 day post challenge which started to recover on days 4–6, at a faster rate than in the placebo mice. Interestingly, the NS1 mRNA-LNP group started losing weight on day 8 and recovered entirely by the end of the study on day 14.

On day 6 after challenge, serum viral RNA (vRNA) loads were determined by quantitative reverse transcription polymerase chain reaction (qRT-PCR). Compared to placebo-treated animals, all vaccinated mice showed substantially reduced (*p* < 0.0002) viral copy numbers suggesting reduced viral replication (Fig. 1b). The lowest viral loads were found in the groups immunized with prM-E mRNA-LNP vaccine either alone or in combination with the NS1 mRNA-LNP. Most animals receiving only the NS1 mRNA-LNP vaccine had a similar level of vRNA copies to the prM-E mRNA-LNP groups, but two animals developed high viral loads comparable to those in the placebo group suggesting breakthrough infections.

Humoral immune responses to vaccination and infection were determined using 50% focus reduction neutralization test (FRNT_50_) to evaluate nAbs and enzyme-linked immunosorbent assay (ELISA) to quantify NS1-binding antibodies. Three weeks after the first vaccination, mice vaccinated with 5 µg of the bivalent prM-E/NS1 mRNA-LNP vaccine showed similar FRNT_50_ levels as mice in the YF-17D group (Fig. 1c). Furthermore, dose-dependency was observed for the prM-E mRNA-LNP vaccine, showing a geometric mean titer (GMT) of 287 for mice receiving 2.5 µg, 124 for mice receiving 0.5 µg of prM-E mRNA alone, and 156 for mice receiving 0.5 µg each of prM-E and NS1 mRNAs. As expected, no neutralization activity was detected after vaccination with 4 μg NS1 mRNA-LNP since NS1-specific antibodies are non-neutralizing. A second dose of the mRNA-LNP vaccines increased FRNT_50_ titers markedly. Two weeks after the second immunization (week 5), both groups immunized twice with 0.5 µg prM-E mRNA-LNP showed high FRNT_50_ levels with GMT of 3662 for mice receiving 0.5 µg of prM-E mRNA-LNP alone, and 4688 for mice receiving 1 µg of the bivalent prM-E/NS1 mRNA-LNP. Interestingly, YFV challenge had no boosting effect on FRNT_50_ levels in the groups immunized with the monovalent prM-E or bivalent prM-E/NS1 mRNA-LNP vaccines suggesting protective immunity provided by vaccination. The GMT of the groups immunized with two doses of the 0.5 µg prM-E mRNA-LNP vaccine decreased after challenge (*p* < 0.0073), while no variation in the GMT was observed for the groups immunized once with the bivalent prM-E/NS1 mRNA-LNP or the YF-17D vaccine. In contrast, high FRNT_50_ levels were detected after challenge in the placebo group (GMT 1314; *p* < 0.0001) and for two animals receiving the YF NS1 mRNA-LNP vaccine. The latter were the same animals that showed high viral loads, confirming breakthrough infections in these mice.

YFV NS1-binding antibody levels were comparable in mice vaccinated with a single 5 µg dose of the bivalent prM-E/NS1 mRNA-LNP vaccine or the YF-17D vaccine 3 and 5 weeks after the first immunization (Fig. 1d). Furthermore, a dose effect was observed after the first dose for the NS1 response in groups receiving a total dose of 1 or 5 µg of the bivalent prM-E/NS1 mRNA-LNP, which did not increase with a 4 µg NS1 only dose. Mice immunized a second time with 4 µg NS1 or 1 µg of the prM-E/NS1 mRNA-LNP showed an increase in antibody levels and comparable GMTs at week 5, showing little to no effect of the amount of the vaccine on antibody levels at that point. As expected, mice receiving the prM-E mRNA-LNP vaccine alone showed no detectable YFV NS1-binding antibodies. After challenge, the GMT of the group immunized with two doses of 0.5 µg each of the prM-E and NS1 mRNA-LNPs showed no further increase, while anti-NS1 antibody levels increased in all other groups. Importantly, the same two mice from the 4 µg NS1 mRNA-LNP vaccine group which showed high viral loads and nAb levels also developed high anti-NS1 antibodies after challenge.

Taken together, although all mice survived the challenge due to age-dependent resistance, signs of infection and YF disease could be detected in placebo-treated animals, which were reduced or absent in vaccinated animals suggesting that mRNA-LNP vaccines can provide protection.

### Serum from vaccinated mice protects from lethal challenge via passive immunity

**Fig. 2:**
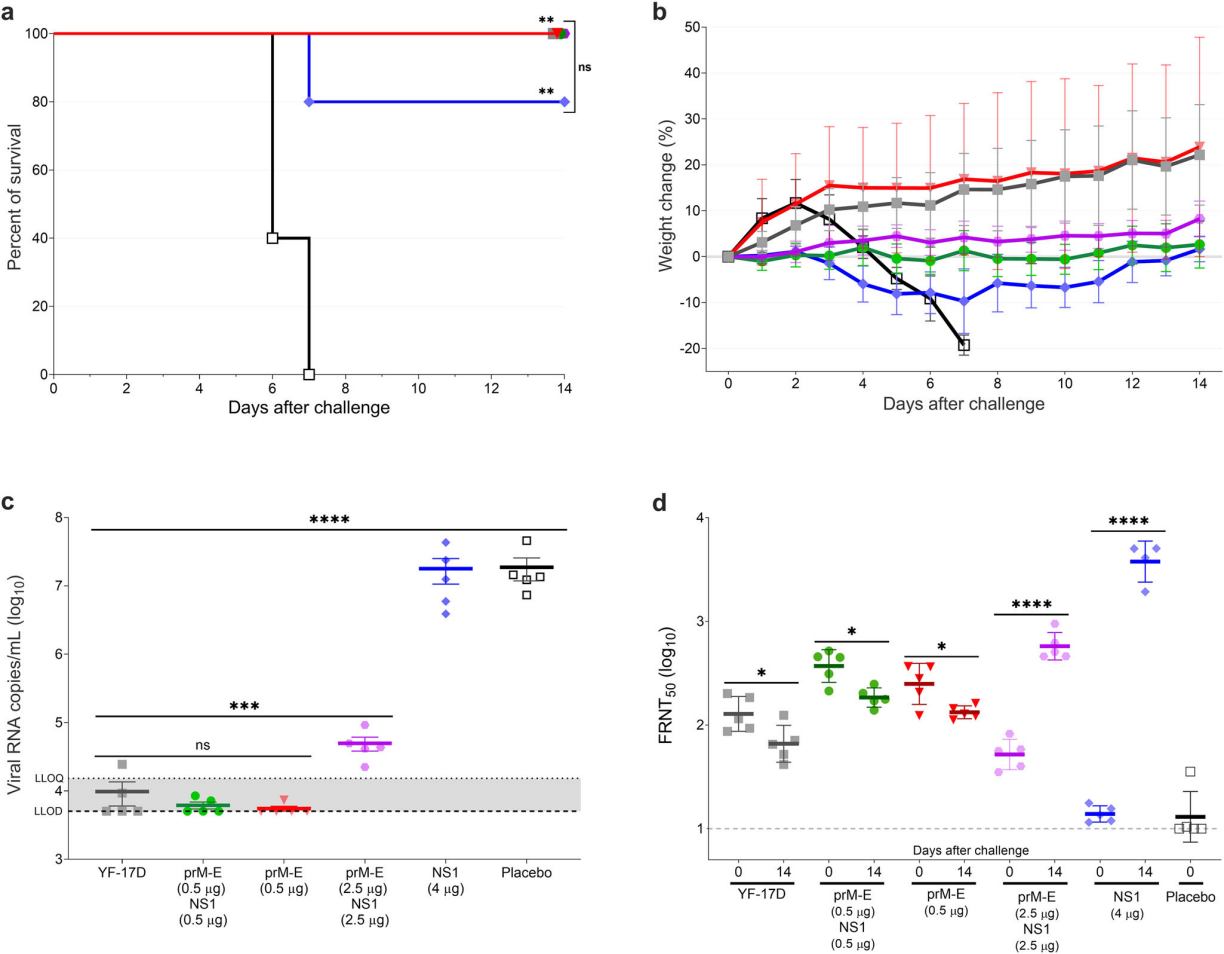
Passive transfer of immune sera protects from lethal YFV challenge in mice. Three- to four-week-old A129 mice (*n* = 5) received sera from vaccinated groups: one dose of one-tenth of the human dose of YF-17D vaccine (close square; gray) or bivalent YF prM-E and NS1 mRNA-LNPs (2.5 µg each) (hexagon; violet), two doses of 0.5 µg of YF prM-E mRNA-LNP (inverted triangle; red), bivalent YF prM-E and NS1 mRNA-LNPs (0.5 µg each) (circle; green), 4 µg of YF NS1 mRNA-LNP (diamond; blue), or placebo group (open square; black). Sixteen hours later, mice were bled and infected IP with 1 × 10^5^ PFU of YFV Asibi strain. Panel **a** shows the survival curve for all groups up to 14 days after the lethal challenge. Panel **b** shows the percentage of the body weight change (±SD) of the animals over a period of 14 days post challenge. **c** Serum viral load (±SEM) measured 6 days after challenge by qRT-PCR. Panel **d** shows the neutralizing antibody titers measured by FRNT_50_ (GMT ± GSD). Dashed line shows the lower limit of detection (LLOD), and dotted line shows the lower limit of quantification (LLOQ), *p < 0.05, **p < 0.01, ***p < 0.001, ****p < 0.0001, ns = not significant. The significance of the survival rates was assessed by Log-rank test, one-way ANOVA followed by Dunnett’s multiple comparisons test or two-way ANOVA followed by Šidák’s multiple comparisons test was performed using GraphPad Prism for serum viral load and neutralizing antibody titers, respectively. These tests were performed after the data was log-transformed and assessed for normality using a Q–Q plot.

To evaluate the protective role of the humoral immune responses elicited by the prM-E and NS1 mRNA-LNP vaccine candidates in more detail, we performed a passive transfer experiment in naive 3–4-week-old A129 mice using serum from vaccinated adult animals from study 1 (Supplementary Fig. 1b). Six groups of young recipient mice (*n* = 5 per group) received 200 µL of serum per animal from vaccinated A129 mice. Pre-challenge sera collected in the vaccination experiment in weeks 3, 4, and 5 were pooled and injected via intraperitoneal (IP) injection. Twelve hours after the passive immunization, recipient mice were bled, challenged IP with 1 × 10^5^ PFU of YFV Asibi strain, and monitored daily for 2 weeks. The inoculation route and infectious dose were adapted compared to study 1 to increase morbidity in A129 mice after YFV challenge. All recipient mice of serum derived from placebo controls showed clinical signs of YF disease displayed as piloerection, hunched posture, lethargy, or orbital tightening and were euthanized 6–7 days after challenge (Fig. 2a). Meanwhile, all recipient mice of serum from mice immunized with the prM-E mRNA-LNP (alone or in combination with NS1 mRNA-LNP) and the YF-17D vaccine survived the challenge (Fig. 2a) and showed no signs of body weight loss (Fig. 2b). Mice which received the serum from the 4 µg NS1 mRNA-LNP immunized group were partially protected (survival 4/5) (Fig. 2a) but displayed piloerection, lethargy, and body weight loss that resolved by the end of the study (Fig. 2b). Immune sera from the two-dose 0.5 µg prM-E mRNA-LNP and bivalent prM-E/NS1 mRNA-LNP (0.5 µg each) vaccinated groups (*p* < 0.0001), reduced serum vRNA copies to undetectable levels on day 6 after challenge (Fig. 2c). In line with that, FRNT_50_ levels in these groups decreased on day 14 after challenge (*p* = 0.0113 and *p* = 0.0269, respectively, Fig. 2d). Immune serum from one-dose YF prM-E/NS1 mRNA-LNP (2.5 µg each) vaccinated mice substantially decreased viral loads compared to placebo sera-recipient mice (*p* < 0.0001, Fig. 2c) and induced a moderate increase of FRNT_50_ (*p* < 0.0001), suggesting very low levels of viral replication remained. In contrast, serum from two-dose immunized 4 µg NS1 mRNA-LNP mice (Fig. 2d) had no effect on viral loads and strong induction of FRNT_50_ levels was observed 2 weeks after challenge, triggered by high levels of viral replication. In summary, animals receiving immune serum from mice immunized with two doses of prM-E mRNA-LNP were protected from morbidity and showed minimal virus replication after challenge. Animals receiving immune serum derived from single dose bivalent prM-E and NS1 mRNA-LNP vaccinated mice had detectable virus load and showed production of nAbs in response to virus replication. Although the 4 µg NS1-mRNA-LNP immune serum-recipient mice had high viral loads and FRNT_50_ titers, the NS1-specific antibodies protected 4/5 mice from an otherwise lethal YFV challenge. The passive serum transfer data differs from the active vaccination study where most NS1 vaccinated mice had low serum viral load and low FRNT_50_ titers after challenge (Fig. 1b, c). This suggests immune effectors other than serum antibodies, e.g., T cells, could contribute to the control of YFV infection in these mice.

### Cell-mediated immunity induced by immunization with YF mRNA vaccines protects A129 mice after adoptive transfer

**Fig. 3:**
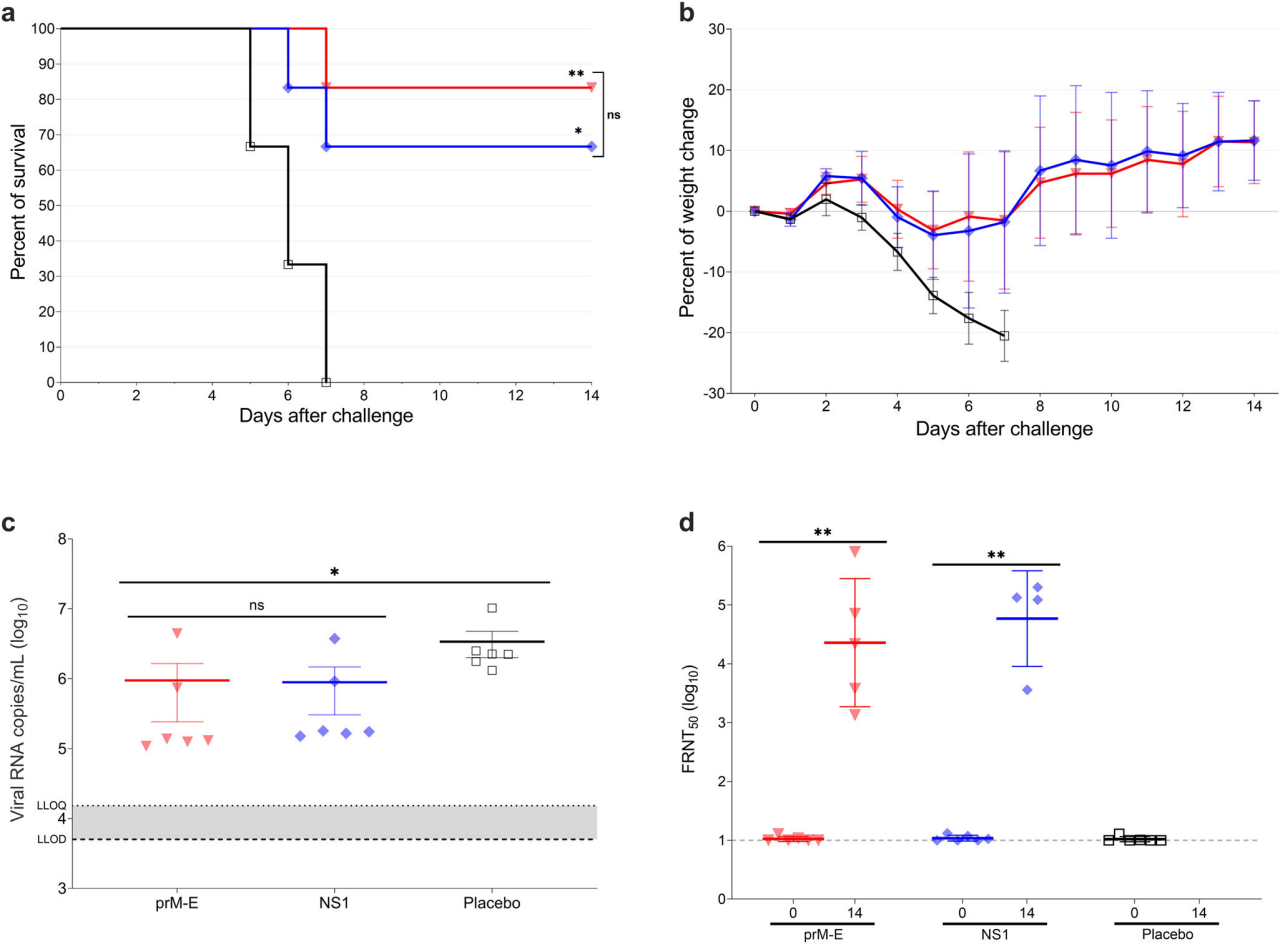
Adoptive transfer of immune-splenocytes provides partial protection against lethal YFV challenge in A129 mice. Three- to four-week-old A129 mice (*n* = 6) received splenocytes from vaccinated groups: 4 µg of YF prM-E mRNA-LNP (inverted triangle; red), 4 µg of YF NS1 mRNA-LNP (diamond; blue), or placebo (open square; black). Sixteen hours later, mice were bled and infected IP with 1 × 10^5^ PFU of YFV Asibi strain. Panel **a** shows the survival curve during 14 days after lethal challenge. Panel **b** shows the percentage of the body weight change (±SD) of the animals over a period of 14 days after the challenge. **c** Serum viral load (±SEM) measured 3 days after the challenge by qRT-PCR. Panel **d** shows the neutralizing antibody titers measured by FRNT_50_ (GMT ± GSD). Dashed line shows the lower limit of detection (LLOD), and dotted line shows the lower limit of quantification (LLOQ), *p < 0.05, **p < 0.01, ***p < 0.001, ****p < 0.0001, ns = not significant. The significance of the survival rates was assessed by Log-rank test, one-way ANOVA followed by Dunnett’s multiple comparisons test or two-tailed *t*-test was performed using GraphPad Prism for viral load and neutralizing antibody titers, respectively. This test was performed after the data was log-transformed and assessed for normality using a Q–Q plot.

To determine if YF mRNA-LNP vaccines protect mice via T cell-mediated immunity, we performed an adoptive transfer experiment using splenocytes isolated from vaccinated A129 mice. First, three groups of 7–9-week-old mice (*n* = 6 per group) were inoculated with 4 µg of prM-E mRNA-LNP, 4 µg of NS1 mRNA-LNP, or NaCl buffer (placebo control). The mice were vaccinated twice 3 weeks apart and sacrificed for splenocyte isolation 10 days after the second dose. Next, three groups of 3–4-week-old recipient A129 mice (*n* = 6 per group) received 1 × 10^7^ splenocytes per mouse from vaccinated A129 mice via retro-orbital injection (Supplementary Fig. 1c). Sixteen hours after the passive immunization, all mice were bled, challenged with 1 × 10^5^ PFU of YFV Asibi strain via IP injection, and monitored daily for 2 weeks. The placebo splenocyte-recipient mice showed clinical signs of YF disease as piloerection, hunched posture, lethargy, or orbital tightening and were euthanized 5–7 days after challenge (Fig. 3a). Most of the mice that received splenocytes from the prM-E mRNA-LNP or NS1 mRNA-LNP vaccinated animals survived the challenge (survival of 5/6 and 4/6 animals, respectively). Mice that received immune-splenocytes of prM-E mRNA-LNP and NS1 mRNA-LNP vaccinated animals displayed lethargy and minimal body weight loss that resolved by the end of the study (Fig. 3b). Compared to placebo-treated mice, serum viral loads measured 3 days after challenge showed that the recipients of immune-splenocytes of prM-E mRNA-LNP (*p* = 0.0158) and NS1 mRNA-LNP vaccines (*p* = 0.0227) showed minimal reduction of the vRNA copies (Fig. 3c). High FRNT_50_ titers were detected after challenge on day 14 for the prM-E mRNA-LNP and NS1 mRNA-LNP immune-splenocyte-recipient mice (Fig. 3d). Altogether, most animals receiving prM-E mRNA-LNP and NS1 mRNA-LNP immune-splenocytes survived the lethal challenge, demonstrating the involvement of T cells in controlling YFV infection in this mouse model.

### Two-dose immunization with prM-E and NS1 mRNA-LNP vaccines elicits robust immune responses in rhesus macaques

**Fig. 4:**
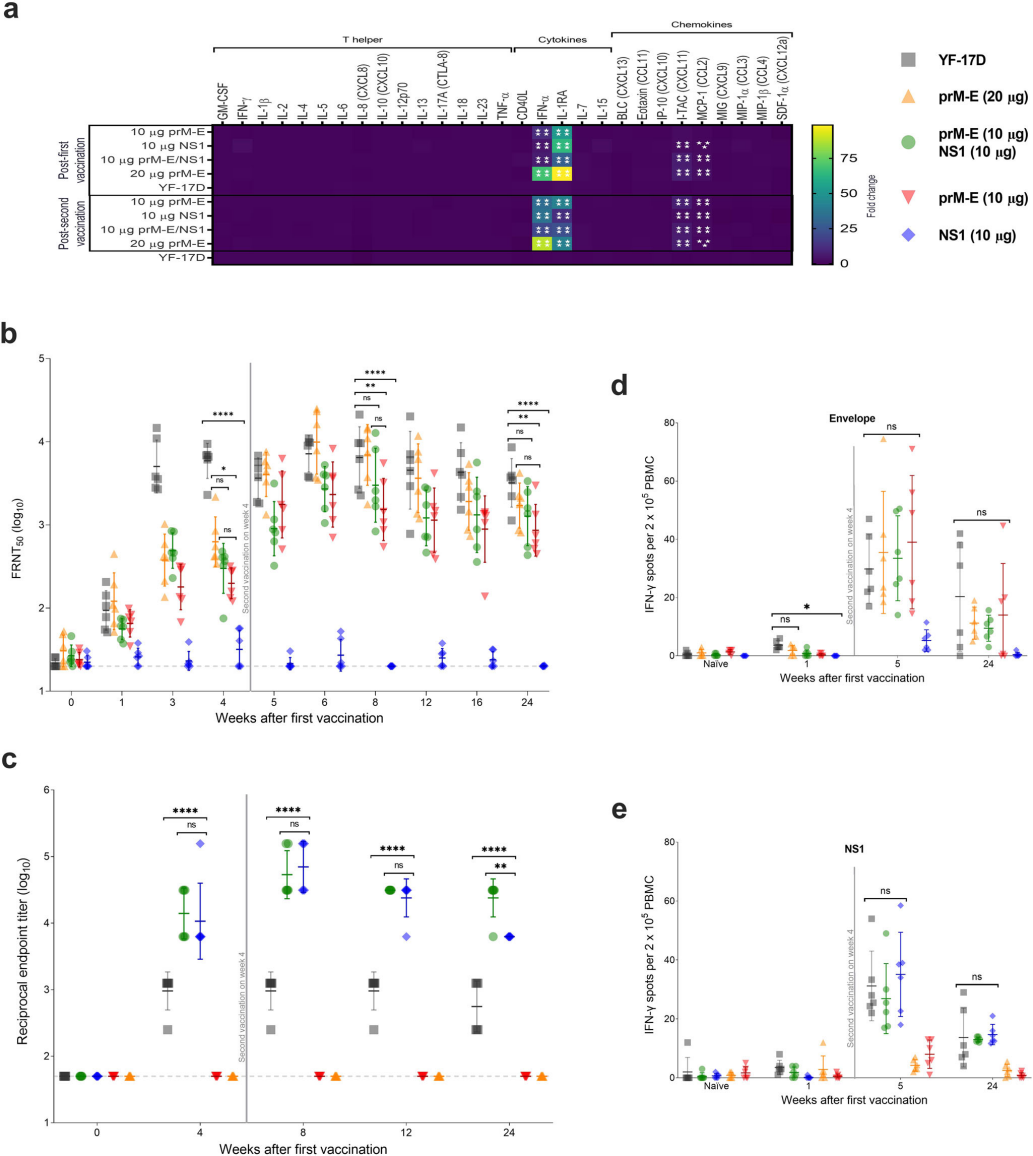
YF mRNA vaccines elicit humoral and cellular immune responses in rhesus macaques. Rhesus macaques (*n* = 6 per group) were immunized once with one full human dose of the YF-17D vaccine (closed square; gray), or two doses of 20 µg YF prM-E mRNA-LNP (triangle; orange), 10 µg YF prM-E mRNA-LNP (inverted triangle; red), 10 µg YF NS1 mRNA-LNP (diamond; blue), or bivalent YF prM-E and NS1 mRNA-LNPs (10 µg each) (circle; green) 4 weeks apart. **a** Heatmap depicts the mean fold change of the levels of cytokines and chemokines in the NHP serum 16 h after each vaccination compared to serum collected on day 0 or day 28, respectively. Panel **b** shows the neutralizing antibody titers measured by FRNT_50_ (GMT ± GSD) with selected statistical comparisons. **c** ELISA data showing the YFV NS1-specific antibody titers (GMT ± GSD). **d**, **e** IFN-γ ELISpot responses from naive and vaccinated macaques after stimulation with virus-derived peptides (E and NS1 proteins), expressed as spot-forming cells per 2 × 10^5^ PBMCs. Vertical line indicates time of second immunization, dashed line shows the limit of detection, *p < 0.05, **p < 0.01, ***p < 0.001, ****p < 0.0001, ns = not significant. One-way ANOVA followed by Dunnett’s multiple comparisons test was used for the IFN-γ ELISpot responses. Two-way ANOVA followed by Tukey’s multiple comparisons test for serum cytokine profile. Two-way ANOVA followed by Tukey’s and Dunnett’s multiple comparisons test for neutralizing antibody titers and ELISA data. These tests were performed using GraphPad Prism after the data were log-transformed and assessed for normality using a Q–Q plot.

To analyze the immune responses induced by YF mRNA vaccines in an animal model closely related to humans, we next immunized NHPs (i.e., rhesus macaques) with prM-E mRNA-LNP and NS1 mRNA-LNP vaccines. Five groups of rhesus macaques (*n* = 6 per group, 2–3 years old, mixed sex) were injected IM twice 28 days apart with 10 or 20 µg of the prM-E mRNA-LNP, 10 µg of the NS1 mRNA-LNP, or a bivalent approach with 10 µg of each mRNA-LNP. Control animals were vaccinated once with a full human dose of the YF-17D vaccine (Supplementary Fig. 1d). After vaccination, no inflammation at the injection site was observed. To further evaluate the potential of adverse events induced by the vaccine, we characterized the systemic cytokine and chemokine responses induced after the first and second vaccination, since some cytokines could be associated with adverse events in humans^24^. A panel of 29 cytokines and chemokines was analyzed from the serum of the rhesus macaques before and 16 h after each immunization with the YF mRNA or YF-17D vaccines. Most of the 29 analytes measured on day 1 or 29 showed no significant alteration compared to baseline (day 0 or 28, respectively) (Fig. 4a). After the first and second vaccinations, rhesus macaques receiving the mRNA vaccines, showed increased levels of IFN-α and IL1-RA (*p* < 0.0001) (Fig. 4a). This is in line with previous mRNA vaccination studies where the upregulation of type I IFNs and the subsequent production of IL1-RA have been reported^25,26^. Compared to the first vaccination, the second vaccination induced the production of the chemokines I-TAC (CXCL11) and MCP-1 (CCL2).

nAbs were detected as early as 1 week after the first immunization in the 20 µg prM-E mRNA-LNP (*p* = 0.0012) and YF-17D (*p* = 0.0004) vaccine groups (Fig. 4b). Before the second vaccination at 4 weeks, macaques receiving 10 µg of the prM-E mRNA-LNP vaccine either alone or in combination with NS1 mRNA-LNP showed comparable FRNT_50_ levels. Animals receiving 20 µg of the prM-E mRNA-LNP vaccine showed slightly higher FRNT_50_ levels suggesting a dose effect. Starting from week 5, the 20 µg prM-E mRNA-LNP and YF-17D vaccine groups had similar FRNT_50_ titers, whereas groups receiving 10 µg of prM-E mRNA-LNP either alone or together with NS1 mRNA-LNP in the bivalent vaccine showed somewhat lower FRNT_50_ albeit this was rarely statistically significant (Fig. 4b). As expected, no neutralization activity was observed in animals receiving 10 μg NS1 mRNA-LNP alone.

YFV NS1-specific antibodies were detected 4 weeks after the first immunization and were increased after the second vaccination with the mRNA-LNP vaccines (Fig. 4c). NS1-specific antibody titers were comparable in both groups receiving the NS1 mRNA-LNP vaccine (either alone or together with the prM-E vaccine) at all analyzed timepoints. Importantly, the animals immunized with the YF mRNA-LNP vaccines showed significantly increased anti-NS1 antibody levels compared to the YF-17D group (*p* < 0.0001) throughout the study (Fig. 4c).

Next, we analyzed the induction of cellular immune responses against YFV proteins in peripheral blood mononuclear cells (PBMC) collected prior to vaccination, 1 week after the first (week 1) and second (week 5) immunization as well as 6 months after the study start. Cells were stimulated with peptide libraries spanning the YFV envelope (Fig. 4d) or NS1 (Fig. 4e) proteins and IFN-γ secreting cells were measured by enzyme-linked immunosorbent spot (ELISpot). IFN-γ positive spots were readily detected 1 week after the second immunization. At this timepoint, all animals vaccinated with prM-E mRNA-LNP vaccine had developed E-specific T cells with levels comparable to those induced by the YF-17D vaccine (Fig. 4d). Similarly, NS1 mRNA-LNP vaccinated animals developed IFN-γ secreting T cells specific for YFV NS1 (Fig. 4e). The T cell responses were still detectable in most animals 5 months after the second immunization.

In essence, the two-dose vaccination strategy using YF mRNA-LNP vaccine candidates induced high humoral and cellular immune responses that were comparable to (nAb titers and T cell responses) or exceeded (NS1-binding antibodies) those induced by the licensed YF-17D vaccine in the rhesus macaque model. Importantly, the induced immune responses persisted through the 5 months observation period after the second dose, indicating the induction of lasting humoral and cellular immune responses associated with protection against YFV infection.

### Passive transfer of rhesus macaque immune sera protected mice from lethal challenge

**Fig. 5:**
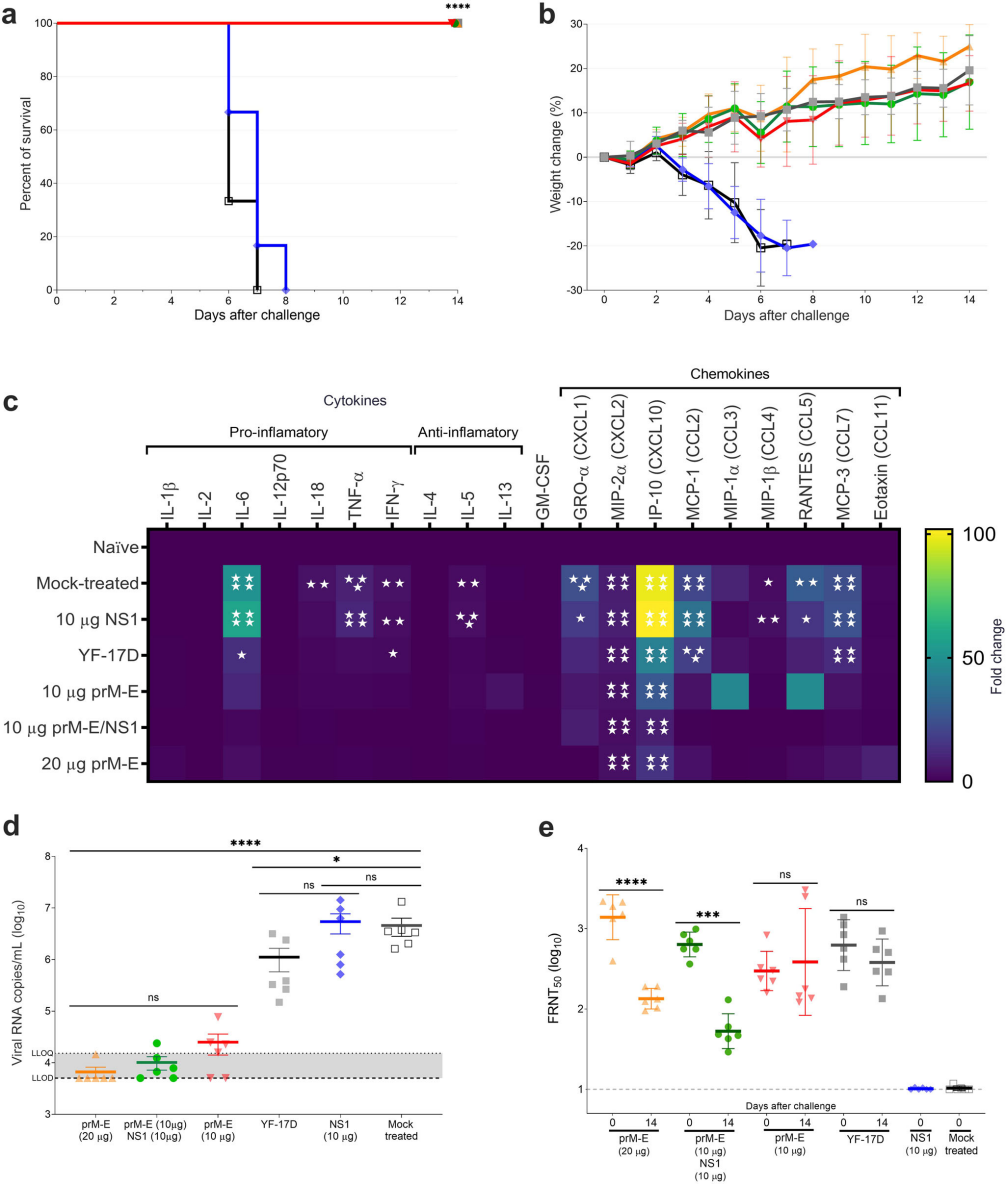
Passive transfer of immune serum from vaccinated macaques can protect from lethal YFV challenge in young A129 mice. Three- to four-week-old A129 mice (*n* = 6) received either naive sera (mock-treated; open square; black), or sera from week 8 of the macaque immunization study: one dose of YF-17D vaccine (close square; gray), two doses of 20 µg YF prM-E mRNA-LNP (triangle; orange), 10 µg YF prM-E mRNA-LNP (inverted triangle; red), 10 µg YF NS1 mRNA-LNP (diamond; blue), bivalent YF prM-E and NS1 mRNA-LNPs (10 µg each) (circle; green). Sixteen hours later, mice were bled and infected IP with 1 × 10^5^ PFU of YFV Asibi strain. Panel **a** shows the survival curve and panel **b** shows the percentage of the body weight change (±SD) of the animals over a period of 14 days after the challenge. **c** Heatmap depicts the fold change of the levels of cytokines and chemokines measured 3 days after challenge, compared to uninfected controls (naive). **d** Serum viral load (±SEM) measured by qRT-PCR 3 days after the challenge. Panel **e** shows the neutralizing antibody titers measured by FRNT_50_ (GMT ± GSD). Dashed line shows the lower limit of detection (LLOD), and dotted line shows the lower limit of quantification (LLOQ), *p < 0.05, **p < 0.01, ***p < 0.001, ****p < 0.0001, ns = not significant. All the following tests were performed using GraphPad Prism. The significance of the survival rates was assessed by Log-rank test, one-way ANOVA followed by Dunnett’s multiple comparisons test was performed for serum cytokine profile, one-way ANOVA followed by Tukey’s and Dunnett’s multiple comparisons test for viral load, while a two-way ANOVA and two-tailed *t*-test were used for the neutralizing antibody titers. Cytokine profiles and FRNT_50_ tests were performed after the data were log-transformed and assessed for normality using a Q–Q plot.

To indirectly evaluate the protection mediated by the humoral immune responses elicited through vaccination of NHPs with YF prM-E and NS1 mRNA-LNPs, we performed a passive transfer experiment in naive A129 mice using serum from vaccinated rhesus macaques (Supplementary Fig. 1e). Six groups of 3–4-week-old A129 mice (*n* = 6 per group) received 200 µL per mouse of serum from vaccinated macaques. Sera collected at week –2 (naive serum) or week 8 (immune serum) were injected via IP route and 16 h after the passive transfer, mice were bled, challenged with 1 × 10^5^ PFU of YFV Asibi strain, and monitored daily for 2 weeks. All recipient mice of the naive serum (mock-treated) and serum from NS1 mRNA-LNP immunized animals reached the humane endpoint and were euthanized 6–8 days after challenge (Fig. 5a). These animals showed high body weight loss and clinical signs of YF disease (Fig. 5b). Meanwhile, all mice receiving prM-E mRNA-LNP or YF-17D immune serum survived the challenge and displayed no body weight loss or clinical symptoms (Fig. 5a, b).

YFV infection increases serum cytokine levels in A129 mice^22^. To ascertain whether passive transfer of serum antibodies can prevent the induction of these cytokines after YFV infection, we performed cytokine profiling 3 days after the challenge (Fig. 5c). YFV infection increased levels of IL-6, TNF-α, IFN-γ, IL-5, GRO-α, MIP-2α, IP-10, MCP-1, MIP-1β, RANTES, and MCP-3 in mice treated with serum derived from naive rhesus macaques and those receiving NS1 mRNA-LNP immune serum, whereas cytokine and chemokine levels of IL-1β, IL-2, IL-12p70, IL-4, IL-13, GM-CSF, MIP-1α, and Eotaxin showed no alteration from baseline. Compared to the mock- or NS1-treated group, mice receiving prM-E or YF-17D immune serum showed decreased levels of all cytokines induced by YFV infection, e.g., IL-6 levels in mice receiving any prM-E immune serum at baseline and IP-10 levels were strongly reduced (Fig. 5c).

Serum viral load measurements 3 days after challenge showed that, compared to mock-treated mice, all prM-E mRNA-LNP groups had substantially reduced (*p* < 0.0001) vRNA copies (Fig. 5d), whereas the NS1-immune serum had no effect on virus copy numbers. FRNT_50_ levels confirmed reduced virus activity. nAb titers of most mice receiving any prM-E immune serum were decreased on day 14 after challenge compared to day 0 (16 h after passive transfer) (Fig. 5e). Whereas GMTs of groups receiving immune serum from 10 µg prM-E mRNA-LNP or YF-17D vaccination did not change between these timepoints. This data suggests that passively transferred antibodies from NHPs receiving 20 µg prM-E or the bivalent vaccine of 10 µg prM-E and 10 µg NS1-immune serum provided protective immunity to A129 mice, leading to reduced cytokine and chemokine induction and inhibition of virus replication. FRNT_50_ levels waned over time confirming detection of NHP-derived antibodies. In groups receiving immune serum from 10 µg prM-E mRNA-LNP or YF-17D vaccinated mice, protection from YFV challenge was less pronounced. Some virus replication was still observed, which likely induced low-level nAb production in the mice. This can be detected at 2 weeks post challenge, where a decrease in FRNT_50_ activity is barely detectable. Notably, two mice receiving prM-E immune serum (10 µg group) showed increased FRNT_50_ on day 14 after challenge confirming that murine antibodies are being produced in these mice. Importantly, groups receiving immune serum from prM-E vaccinated NHPs showed a dose effect with an initial nAb GMT = 303 in the 10 µg prM-E group with signs of virus replication and GMT = 1338 in the 20 µg prM-E group with only one animal showing serum viral loads above the limit of detection.

## Discussion

The current COVID-19 pandemic and the 2013–2016 outbreak of the Ebola virus exposed the global lack of preparation to confront these outbreaks. Collective efforts such as the ones pursued by the Coalition for Epidemic Preparedness Innovations (CEPI) to stimulate and accelerate the development of vaccines are urgently needed. Due to increasing population density, ecological change, and human mobility, re-emerging and emerging infectious diseases pose an increasing threat to humanity.

Another example is the recent YFV outbreak in Brazil. It raised serious concerns due to its rapid dissemination between 2016 and 2019^27,28^. Fortunately, a single shot of the YF-17D vaccine can provide lifelong protection against YF by eliciting a robust immunological response that includes both the innate and adaptive arms of the immune system^29^. Nevertheless, it comes with its own risks and shortcomings^12^. Although the global YF vaccine manufacturing capacity is increasing, supported by the Eliminate Yellow Fever Epidemics strategy^13^, it would be inadequate to contend with a serious outbreak^30^. Large YF outbreaks rapidly exhaust the emergency vaccine stockpiles, limiting the availability and accessibility of YF vaccines^31^. As a result, fractional dosing studies for the licensed YF vaccines are under phase 4 clinical trials^32–34^. These studies aim to determine the lowest dose needed for YF prevention, which can subsequently afford dose-sparing strategies in response to large outbreaks. To overcome vaccine scarcity, and as part of CEPI’s vaccine development program, we produced and tested mRNA vaccine candidates against YF.

Here, we present pre-clinical data for two highly effective mRNA vaccine candidates: the YF prM-E mRNA-LNP vaccine that encodes the prM-E polyprotein and the YF NS1 mRNA-LNP vaccine that encodes the NS1 protein. These vaccine candidates are based on the RNActive^®^ platform (described in, e.g., WO2002098443 and WO2012019780). A two-dose immunization regimen of the prM-E mRNA-LNP vaccine candidate induced robust immune responses in mouse and macaque models with nAb titers and T cell responses comparable to those seen with the licensed YF-17D vaccine. Importantly, a single vaccination with a bivalent YF prM-E and NS1 mRNA-LNP vaccine candidate also induced high-level immune responses and conferred protection from lethal YFV challenge in passive transfer studies. In macaques, the nAbs and T cells induced by the mRNA-LNP vaccines were stable for at least 5 months after the second immunization, indicating the induction of lasting immune responses associated with protection against YFV infection. Evaluation of the cytokine and chemokine responses in the serum of vaccinated macaques showed that the cytokines IFN-α and IL1-RA and chemokines I-TAC and MCP-1 were predominantly upregulated. Type I IFN (IFN-α) is naturally produced in response to exogenous unmodified mRNA and consequently leads to T_H_1 responses^25^. Other cytokines associated with T_H_1 (IFN-γ, IL-2, IL-12, and TNF-α) or T_H_2 (IL-5, IL-6, IL-10, and IL-13) responses were not detected at 16 h after either vaccination. Notably, IL-4 levels were either very low or undetectable after mRNA-LNP vaccination, indicating a vaccine profile that avoids the establishment of T_H_2-bias and the possible exacerbation of infection or counteracting T_H_1 responses^35^.

We performed a series of passive transfer studies to further characterize the protection induced by prM-E and NS1 mRNA-LNP vaccine candidates in a lethal challenge model. Before the challenge, young naive A129 mice were treated with immune serum or splenocytes from prM-E or NS1 vaccinated mice. Both the serum (100% survival for prM-E and 83% survival for NS1) and the cells (80% survival for prM-E and 66% survival for NS1) conferred protection, while naive mice receiving non-immune serum or splenocytes succumbed to the challenge. When we passively transferred macaque immune serum, all mice receiving the prM-E-immune serum survived the lethal challenge suggesting nAbs in the serum were sufficient to prevent infection. All mice receiving the NS1-immune serum from macaques had to be euthanized, although NS1-immune serum from mice partially protected from YF challenge. This data suggests that the species of the immune serum plays an important role in whether it can confer protection from YFV infection after passive transfer. Thus, non-nAbs against NS1 with Fc-dependent functions could play a role in combatting YFV infection by clearing the virus, e.g., via antibody-dependent cellular cytotoxicity (ADCC) or complement-dependent pathways^36–38^. Previous studies revealed that the protection given by NS1-specific antibodies against flavivirus infection is consistent with the involvement of an FcR-dependent protective mechanism and ADCC is a known mechanism of protection in mice immunized with Zika virus NS1 protein^37–41^. Although Bailey et al. showed that human monoclonal antibodies targeting Zika virus NS1 protein provided protection against disease in mice^42^, an elevated concentration of monoclonal antibodies was required to protect the mice against viral challenge.

We next evaluated the cytokine profile in YFV-challenged mice. Consistent with flavivirus infection in IFNAR^−/−^ mice and humans^22,23,43,44^, YF challenge in A129 mice induced the hyper-production of IP-10 (CXCL10) which plays a significant role in disease outcome and survival^23^. Moreover, we found that IL-6, TNF-α, IFN-γ, IL-5, GRO-α, MIP-2α, IP-10, MCP-1, MIP-1β, RANTES, and MCP-3 levels were increased in mice with high morbidity or mortality. Notably, passive transfer of prM-E immune serum reduced and, in some cases, even prevented induction of these cytokines and chemokines further highlighting the protection provided by the YF mRNA-LNP vaccine.

Much remains to be learned about the immune responses induced by both YF prM-E and NS1 mRNA-LNP vaccine candidates. Although the prM-E mRNA-LNP is sufficient to confer protection against YF disease, a detailed understanding of how the NS1 mRNA-LNP is protecting mice from a lethal YFV challenge would be beneficial. In our active immunization and challenge study, NS1 immunization led to reduced serum viral loads after challenge, which could be recapitulated in the adoptive transfer study using NS1-immune-splenocytes. Since higher viral loads were recently reported as correlating with higher mortality^45^, it is plausible that NS1-specific T cells could contribute to protection from YF by limiting the number of virus-infected cells. In general, the pre-clinical findings presented in this publication strongly support that the prM-E mRNA-LNP and the NS1 mRNA-LNP vaccine candidates are highly immunogenic with a protective profile. Further investigation of the long-term immunity and a deeper understanding of the T cell responses and functionality of the antibody response induced by these mRNA vaccine candidates are needed.

Until now, four different vaccine platforms comprising at least 10 YF vaccine candidates have been reported in the literature^46,47^. Currently, the inactivated XRX-001 and the recombinant viral vector MVA-BN-YF are the most advanced (phase 1 clinical trial) YF vaccine candidates^48–51^. These vaccine candidates aim to produce safer or more scalable platforms for YF vaccine production. In macaques, a two-dose regimen of XRX-001 induced higher nAbs (GMT = 1016; *n* = 3) than a single dose of YF-17D (YF-VAX^®^, Sanofi Pasteur; GMT = 640; *n* = 2), but a rapid decrease of the nAb titers was observed^49,52^. Although XRX-001 represents a safer alternative to live-attenuated YF-17D vaccines, it needs to be produced in cell culture, purified, inactivated, and adsorbed with aluminum hydroxide (alum) adjuvant. Therefore, this platform requires rather complex manufacturing processes. Similarly, prime-boost MVA-BN-YF vaccination in hamsters induced comparable nAb titers to YF-VAX^®^^51^. This platform appears safe, is highly immunogenic^53–56^, and could potentially expand the global YF vaccine supply.

Here we demonstrated that mRNA vaccine technology could match or even outcompete a licensed YF vaccine. High levels of antibodies and T cells were induced after a two-dose vaccination schedule with the YF prM-E and NS1 mRNA-LNP vaccine candidates. However, all doses and both, single- or two-dose, vaccination strategies of the YF prM-E mRNA-LNP vaccine candidate tested induced strong humoral immune responses that protected mice from a lethal challenge. Our data indicate similar correlates of protection for the YF mRNA-LNP vaccines and YF-17D and support future non-inferiority trials in humans. While YF-17D has the advantage of being highly immunogenic after a single vaccination, a two-dose regimen of an mRNA-LNP vaccine could make up for this with a better safety profile. This would be especially important for at-risk populations for which YF-17D is contraindicated, such as pregnant women, infants, seniors, immunocompromised individuals, and people with hypersensitivity to the YF-17D vaccine components. In addition, mRNA vaccines use a faster and flexible manufacturing process. The mRNA-based vaccine technology enables the production of diverse vaccines using the same standardized processes, equipment, and facilities, therefore making it suitable for large-scale, rapid, and cost-effective vaccines. Thus, a YF vaccine capable of meeting a broader demand, could give people in endemic regions access to the vaccine before outbreaks occur.

## Methods

### Cells and viruses

HeLa (ATCC: CCL2) and Vero (ATCC: CCL-81) cells were cultured in growth media (Dulbecco’s modified Eagle media [DMEM], 10% fetal bovine serum [FBS], and antibiotics), and incubated at 37 °C and 5% CO_2_.

YFV BeH 622205, Asibi, and YF-17D-204 strains were amplified by inoculating confluent Vero cells with a multiplicity of infection of 0.01 and were stored at –80 °C until use. Viral titer was determined by plaque assay. YFV Asibi strain consistently produced higher viral titers than strain BeH 622205. Of note, when used at the same dose, YFV Asibi and BeH 622205 strains induced similar clinical signs in A129 mouse challenge experiments (Supplementary Fig. 2) suggesting no difference in pathogenicity between the strains.

### Plaque assay

To determine the viral titer, a plaque assay was implemented and expressed as PFU per milliliter (PFU/mL). Vero cells seeded in 6-well plates were infected with 200 µL of 10-fold dilution (10^–2^–10^–6^) of the viral stock, incubated at 37 °C for 4 h, and shaken every 30 min. After infection, 3 mL per well of overlay solution (DMEM with 1% carboxymethyl cellulose and 2% FBS) was added and plates were incubated at 37 °C and 5% CO_2_. Seven days post infection, the overlay was discarded, plates were washed three times with phosphate buffer saline (PBS), fixed with 2 mL per well of fixing buffer (3.7% paraformaldehyde in PBS), and incubated for 30 min at room temperature (RT). Plates were then washed with PBS and stained with 0.5% crystal violet staining solution for 30 min at RT. After staining, the fixed cells were washed three times with PBS and air dried overnight before counting the number of plaques. The titer was calculated using the formula PFU/mL = (*P* × *D*) / *V*, where *P* is average number of plaques, *D* is dilution factor, and *V* is volume of inoculum.

### mRNA production and transfection

The mRNA vaccine candidates are composed of a 5′ cap structure, a GC-enriched open reading frame, 3′ UTR, polyA tail, and include unmodified nucleosides. LNP-encapsulation of mRNA was performed with Acuitas Therapeutics LNP technology (Vancouver, Canada). The LNPs were composed of an ionizable amino lipid, phospholipid, cholesterol, and a PEGylated lipid. Two mRNA vaccines were used in this study, one encoding the prM-E polyprotein, the other encoding NS1. Both sequences were derived from the YF-17D vaccine strain (GenBank accession # NC_002031).

Expression of mRNA-encoded YFV proteins was confirmed in HeLa cells. After mRNA transfection, cells expressed and secreted NS1 and formation of virus-like particles by M and E was demonstrated.

### Animal care and ethics statement

All animal studies followed the US National Research Council’s Guide for the Care and Use of Laboratory Animals, the Animal Welfare Act, and the recommendations of the Weatherall report. All animal research was conducted under the authority of the University of Wisconsin-Madison (UW-Madison) School of Veterinary Medicine (SVM) and supervised by the UW-Madison Research Animal Resources and Compliance (RARC). The protocol (# G005519-R01-A01) was approved by the UW-Madison Institutional Animal Care and Use Committee. Rhesus macaques were handled by qualified personnel from the Wisconsin National Primate Research Center (WNPRC) veterinary staff and were free of Macacine herpesvirus 1, simian immunodeficiency virus, simian T-lymphotropic virus type 1, simian retrovirus type D, and *Mycobacterium tuberculosis*. All mouse experiments with the YFV challenge were conducted in animal biosafety level 3 facilities in the Animal Health and Biomedical Sciences building.

### Vaccination of mice

A129 mice of 129/SvEv genetic background were bred in pathogen-free animal facilities at UW-Madison SVM. Mice were randomly allocated to groups and acclimated for 3 days before the initiation of a study. Twelve 7–9-week-old mixed-sex A129 mice were used per group. The vaccines were administered into the *M. tibialis*. For the first mouse study, three treatment groups (*n* = 8 per group) received two vaccinations with the YF prM-E mRNA-LNP (0.5 μg), YF NS1 mRNA-LNP (4 μg), or a bivalent vaccine containing both mRNA vaccines (0.5 μg each) 3 weeks apart or the bivalent vaccine with 2.5 μg each as a single dose. Placebo mice group (*n* = 4) received 0.9% NaCl solution. The positive control group (*n* = 5) received a single vaccination with a tenth of the human dose of the YF-17D vaccine (17D-204, Stamaril^®^; Sanofi Pasteur). Blood draws were performed on week 3 (d20, before second vaccination), week 5 (d34, pre-challenge) to collect serum for serology, and 6 days after challenge (day 42) to measure viral load via qRT-PCR; final bleeding was done on week 7 (day 49) for serological analysis. Additional blood draws on week 4 were pooled with serum from weeks 3 and 5 for further passive immunization experiments (Supplementary Fig. 1a). All sera were equally pooled from each mouse per timepoint and between timepoints to create the pooled serum used for the passive immunization experiments.

A129 mice were challenged IM on week 5 with a dose of 1×10^4^ PFU of YFV BeH 622205 strain in 30 µL of PBS. Animals were monitored daily for 2 weeks for body weight change or signs of disease.

In addition, a vaccination experiment for splenocyte donation was carried out using six 7–9-week-old mixed-sex A129 mice. Three groups of mice (*n* = 6 per group) received two vaccinations of 4 µg of YF prM-E mRNA-LNP, 4 µg of YF NS1 mRNA-LNP, or 0.9% NaCl buffer 3 weeks apart. Mice were sacrificed 10 days after the second vaccination and splenocytes were isolated.

### Vaccination of non-human primates

The groups consisted of three female and three male Indian rhesus macaques (*Macaca mulatta)*, 2–3 years of age, weighing 3–6 kg. Prior to vaccination or bleedings, macaques were ketamine-anesthetized or with a mixture of ketamine/dexmedetomidine with atipamezole reversal and monitored regularly until fully recovered from anesthesia. In brief, macaques were injected IM into the deltoid muscle with 500 µL of mRNA or YF-17D vaccines. mRNA vaccine groups consisted of YF prM-E mRNA-LNP (10 or 20 µg), YF NS1 mRNA-LNP (10 µg), and a bivalent prM-E/NS1 vaccine (10 µg of each mRNA-LNP). The positive control group was vaccinated with a full human dose of the YF-17D licensed vaccine (17D-204, Stamaril^®^, Sanofi Pasteur). One animal from the positive control group (animal r18015) presented diarrhea and *Campylobacter coli* infection and was treated with azithromycin for 5 days before the first vaccination. All mRNA vaccine groups were administered twice at a 4-week interval while the YF-17D vaccine was injected once. After vaccination, the site of injection was monitored for an inflammatory response. Blood draws were performed before the vaccination (weeks –2 and 0), post first vaccination (weeks 1, 3, and 4), post second vaccination (weeks 5, 6, 8, 12, 16, and 24) to collect serum for serology, and for the measurement of cytokines and chemokines before and 16 h after the first and second immunization (Supplementary Fig. 1d).

### Assessment of protection using passive immunization

Two passive transfer experiments were conducted using 3–4-week-old mixed-sex A129 mice. For the passive transfer of immune mouse serum, mice (*n* = 5 per group) were injected with pooled serum from weeks 3, 4, and 5 of the A129 mouse vaccination experiment by IP injection (Supplementary Fig. 1b). For the passive transfer of macaque serum, mice (*n* = 6 per group) were injected with pooled serum from week –2 (naive serum) or individual serum from week 8 (immune serum) of the macaque vaccination experiment by IP injection (Supplementary Fig. 1e).

To determine if vaccination induced protection via cellular immunity, an adoptive transfer experiment was conducted using three groups of 3–4-week-old mixed-sex mice (6 per group). Ten million splenocytes were administered via retro-orbital injection in a volume of 50 µL. Serum samples were collected before challenge or at week 2 after the adoptive transfer (Supplementary Fig. 1c).

Mice were challenged by IP injection of YFV Asibi strain with a dose of 1 × 10^5^ PFU in 50 µL of PBS. The Asibi strain in combination with a higher dose and the IP inoculation route was used to enhance the disease outcome in young A129 mice after wtYFV infection. These changes were prompted by the mild disease outcome observed in adult A129 mice after challenge with a low dose of YFV BeH 622205. Serum viral load was measured on day 3 or 6 after the challenge (Supplementary Fig. 1). Animals were monitored daily for weight change or signs of disease throughout the course of 2 weeks.

### Focus reduction neutralization test

The microneutralization test measured the inhibition of YFV 17D-204 strain entry by the immune serum. The FRNT was performed using 4-fold serial dilutions (starting 1:5 for mice and 1:10 for macaques) of each serum sample to test for neutralization of 200 PFUs of the YFV 17D-204 strain using Vero cells. Serum samples were heat-inactivated by incubation at 56 °C for 30 min, diluted in DMEM with 1% FBS using four-fold dilutions, added to an equal volume of 200 PFUs of the YFV 17D-204 strain, and incubated at 37 °C and 5% CO_2_ for 2 h. Vero cells reaching 95% confluency after 48 h were infected with 50 µL of the inoculum (serum: virus mix) and incubated for 2 h in 96-well plates. Following incubation, the inoculum was removed,100 µL per well of overlay solution (DMEM with 1.5% carboxymethyl cellulose and 2% FBS) was added, and plates were incubated at 37 °C and 5% CO_2_. Forty-eight hours post infection, the overlay was discarded, plates were washed three times with PBS, fixed with 100 µL per well of fixing buffer (75% acetone, 15% methanol, 5% glacial acetic acid, and 5% PBS), and incubated for 10 min at –20 °C. Plates were washed and stored in PBS at 4 °C until immunoassay.

For the immunoassay, 1:2000 dilution of the YFV hyperimmune mouse ascitic fluid (provided by the World Reference Center for Emerging Viruses and Arboviruses at the University of Texas Medical Branch) was used in blocking buffer (PBS, 0.05% tween-20 and 5% powdered milk), followed by 1:3000 diluted horseradish peroxidase-conjugated goat anti-mouse IgG (H+L) (Invitrogen, Cat. No. 31430) as a secondary antibody and developed with chromogen/peroxide substrate. The plates were scanned in the ELISpot plate reader (ImmunoSPOT-Cellular Technology, Cleveland, OH, USA) and counted by the number of foci with the counting function of the software or with Viridot software. Neutralization titers were calculated in GraphPad Prism 9 relative to virus-only controls per plate.

### Enzyme-linked immunosorbent assay (ELISA)

For the detection of serum anti-NS1 IgG antibodies, an in-house ELISA was performed in 96-well microtiter plates (Thermo Scientific, Cat. No. 3455) coated with 100 ng in 100 µL per well of the YF NS1 protein (The Native Antigen Company, Cat. No. YFV-NS1-500). After incubating overnight, plates were washed (1× PBS, 0.05% tween-20) and blocked for 24 h with a blocking buffer (PBS, 0.05% tween-20, and 1% powdered milk), followed by a wash and incubation for 2 h with eight 5-fold serial dilutions of mouse or macaque serum (1:50 primary dilution) in 100 µL of blocking buffer. After washing, plates were incubated for 1 h with 1:5000 diluted horseradish peroxidase-conjugated goat anti-mouse IgG (H+L) (Invitrogen, Cat. No. 31430) or 1:2000 diluted horseradish peroxidase-conjugated goat anti-human IgG (H+L) (Promega, Cat. No. W4031) in 100 µL of blocking buffer, developed with 1-Step™ Ultra TMB-ELISA substrate solution (Invitrogen, Cat. No. 34028), stopped with 450 nm stop solution for TMB substrate (abcam, Cat. No. ab171529) and the absorbance was measured at 450 nm. The absorbance cutoff value was defined as the mean of a naive serum sample for each plate plus three times the SD. The endpoint titer of a sample was defined as the reciprocal of the highest dilution that gave a positive reaction (above the cutoff).

### Detection of viral RNA from blood samples

RNA was extracted from 25 µL of serum, using TRI Reagent^®^ BD (Molecular Research Center, Inc., Cat. No. TB126) and Direct-zol™ RNA MicroPrep (Zymo Research, Cat. No. R2062) according to the manufacturer’s protocol. vRNA load was quantified using qRT-PCR, using the primers YFallF (5’-GCTAATTGAGGTGYATTGGTCTGC-3’) and YFallR (5’-CTGCTAATCGCTCAAMGAACG-3’), and probe YFallP (5’-FAM/ZEN-ATCGAGTTGCTAGGCAATAAACAC-Iowa Black FQ-3’)^57^. The qRT-PCR was performed using the iTaq^TM^ Universal Probes One-Step Kit (Bio-Rad, Cat. No. 1725141) on an iCycler instrument (Bio-Rad, Hercules, CA, USA) and following the protocol recommended by the manufacturer. Quantification was performed using a 20 μL total reaction containing 1X iTaq Universal Probes Reaction Mix, 0.5 μL iScript Reverse Transcriptase, a final concentration of 0.4 μM of each primer and 0.2 μM of the probe, and the final volume completed with RNA. Thermal cycling was performed at 50 °C for 10 min for reverse transcription, followed by 95 °C for 3 min and then 40 cycles of 95 °C for 15 s and 60 °C for 30 s. RNA standard samples were prepared using in vitro transcribed RNA produced in-house and serially diluted tenfold (10^1^–10^8^). The LLOD and LLOQ were calculated based on the standard deviation of the response (S_y_) of the curve and the slope of the calibration curve (S) according to the formula: LLOD = 3.3(S_y_/S) or LLOQ = 10(S_y_/S), whilst undetected samples were assigned the LLOD value.

### Cytokine analysis

Analyte concentrations for all samples were run in duplicate. For macaques, induction of pro-inflammatory cytokines and chemokines was analyzed in 25 μL of serum using the ProcartaPlex NHP Cytokine & Chemokine Panel 30plex (Thermo Fisher Scientific, Cat. No. EPX300-40044-901), measuring the expression of Eotaxin (CCL11), G-CSF (CSF-3), GM-CSF, IFN-α, IFN-γ, TNF-α, IL-10, IL-12p70, IL-13, IL-15, IL-17A (CTLA-8), IL-18, IL-1β, IL-1RA, IL-2, IL-23, IL-4, IL-5, IL-6, IL-7, IL-8 (CXCL8), IP-10 (CXCL10), I-TAC (CXCL11), MCP-1 (CCL2), MIP-1α (CCL3), MIP-1β (CCL4), SDF-1α, MIG (CXCL9), CD40L, and BLC (CXCL13). The analyte G-CSF (CSF-3) was excluded because of the low number of beads detected (<32).

For mice, cytokine and chemokine levels were quantified 3 days after the challenge. Sera were diluted 1:2, and 25 μL of the dilution was tested using the ProcartaPlex Mouse Th1/Th2 & Chemokine Panel I 20plex (Thermo Fisher Scientific, EPX200-26090-901). Levels of Eotaxin (CCL11), GM-CSF, IFN-γ, IL-1β, IL-12p70, IL-13, IL-18, IL-2, IL-4, IL-5, IL-6, TNF-α, GRO-α (CXCL1), IP-10 (CXCL10), MCP-1 (CCL2), MCP-3 (CCL7), MIP-1α (CCL3), MIP-1β (CCL4), MIP-2α (CXCL2), and RANTES (CCL5) were quantified. Quantifications were performed using a MAGPIX^®^ instrument (Luminex Corp, Austin, TX, USA) according to the manufacturer’s instructions and were analyzed on xPONENT^®^ software v4.2 using a standard curve for each analyte (ProcartaPlex standard mixes). The multianalyte detection of cytokines and chemokines had an LLOQ assay sensitivity. Values assayed below this limit were identified as below the limit of quantification (BLOQ) in the reported data. In addition, the extrapolated concentration values for concentrations were identified as BLOQ. Concentrations that are not detected were identified as “Not Detected” and recorded as BLOQ.

### Enzyme-linked immunosorbent spot (ELISpot)

An ELISpot assay was used to measure IFN-γ secreting PBMCs from naive and vaccinated macaques after stimulation with YFV E and NS1 protein-derived peptides. PBMCs obtained pre-vaccination (week –2), 1 week after each vaccination (weeks 1 and 5), and at the end of the study (week 24) were isolated by using BD Vacutainer^®^ CPT™ Cell Preparation Tube with Sodium Heparin (BD, Cat. No. 362753). PBMCs were collected and washed three times with PBS (Corning, Cat. No. 21-040-CM) by centrifugation at 100 *g* for 10 min. PBS was removed, and cells were resuspended in a freezing medium containing 10% dimethylsulfoxide (DMSO) and 90% FBS and frozen down at –80 °C inside a freezing container overnight to allow gradual and even cooling, followed by storage in liquid nitrogen until the ELISpot assay was performed. In brief, frozen PBMCs were thawed at 37 °C, diluted in X-VIVO 15 serum-free T-cell medium (Lonza, Cat. No. 04-418Q), centrifuged at 100 *g* for 10 min, and resuspended in fresh culture medium. PBMCs were seeded in triplicate (2 × 10^5^ cells/well in 50 μL) into pre-coated IFN-γ ELISpot plates (Mabtech, Cat. No. 3421M-4HPW-10). The cells were incubated with 50 μL of YFV E or NS1 pooled custom peptides (15-mer peptides with 11-mer overlap, Thermo Scientific) at a final concentration of 2 μg/mL of individual peptide, eBioscience™ Cell Stimulation Cocktail (Thermo Fisher Scientific, Cat. No. 00-4970-93) for the positive control, or DMSO for the negative control. PBMCs and stimuli were incubated for 18 h in the presence of 0.5 µg/mL CD28/CD49d co-stimulatory antibodies (BD Fast Immune, Cat. No. 347690). After incubation, cells were washed in washing buffer (PBS with 0.05% Tween-20), incubated with 1 μg/mL biotinylated anti-IFN-γ detection antibody in 100 μL of PBS/0.5% bovine serum albumin (Sigma, Cat. No. A5611) for 2 h at RT, followed by incubation with Streptavidin-HRP (1:1000) for 1 h at RT. The spots of IFN-γ-secreting cells were developed using the TMB substrate solution, scanned in the ELISpot plate reader (ImmunoSPOT-Cellular Technology, Cleveland, OH, USA), and counted by Viridot software. The results were calculated after subtraction of the negative control background and analyzed in GraphPad Prism 9.

### Statistical analyses

The significance of the survival rates was assessed by Log-rank test, while cytokine profile, IFN-γ ELISpot response, viral load, and ELISA data were assessed by one-way ANOVA. FRNT_50_ titers were assessed by one- or two-way ANOVA. These tests were performed after the data were log-transformed and assessed for normality using a Q–Q plot. All data were analyzed with GraphPad Prism 9.3.0 software for Windows, GraphPad Software, San Diego, California USA, www.graphpad.com. A value of *p* < 0.05 was considered statistically significant and *p < 0.05, **p < 0.01, ***p < 0.001, ****p < 0.0001, ns = not significant.

### Reporting summary

Further information on research design is available in the Nature Research Reporting Summary linked to this article.

## Supplementary information

Supplementary Material

Reporting Summary

## Data Availability

Data supporting the findings of this study are available in this paper, Supplementary information, or are available from the corresponding authors upon request.
