# Supplementary material for: Immunogenicity and protective activity of mRNA vaccine candidates against yellow fever virus in animal models
Source: NPJ Vaccines. 2023 Mar 4;8:31. doi: 10.1038/s41541-023-00629-7 (PMC9984760; doi:10.1038/s41541-023-00629-7)
Supplement: Supplementary file 1 — Supplementary Material [file 41541_2023_629_MOESM1_ESM.pdf]

SUPPLEMENTARY INFORMATION

a

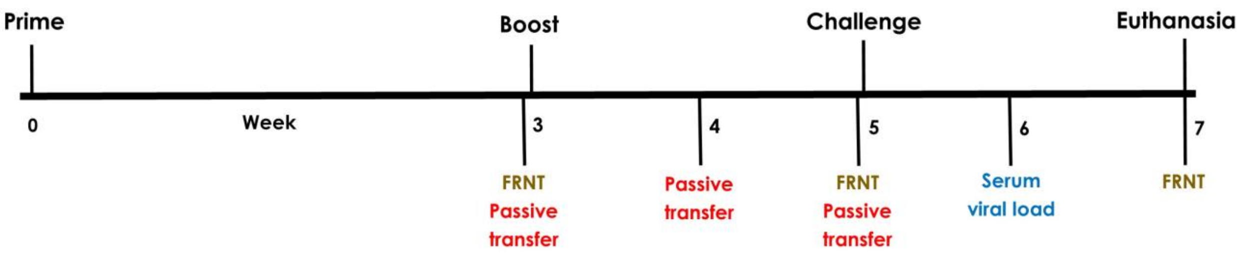

b

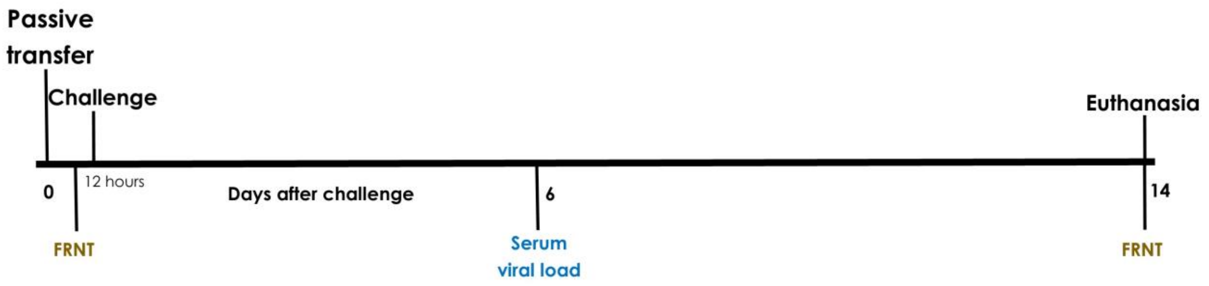

c

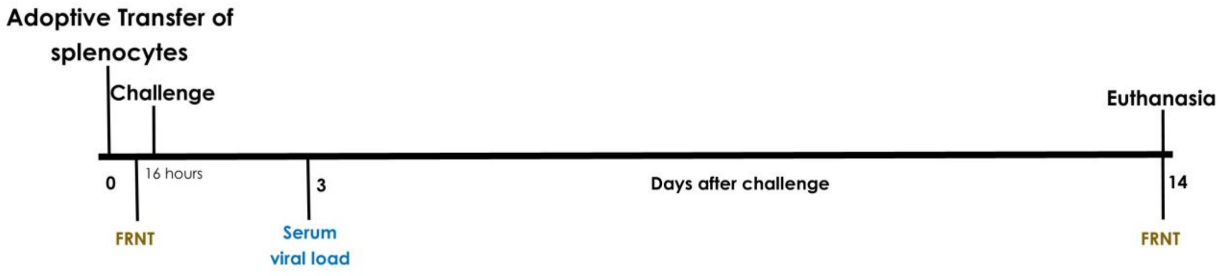

d

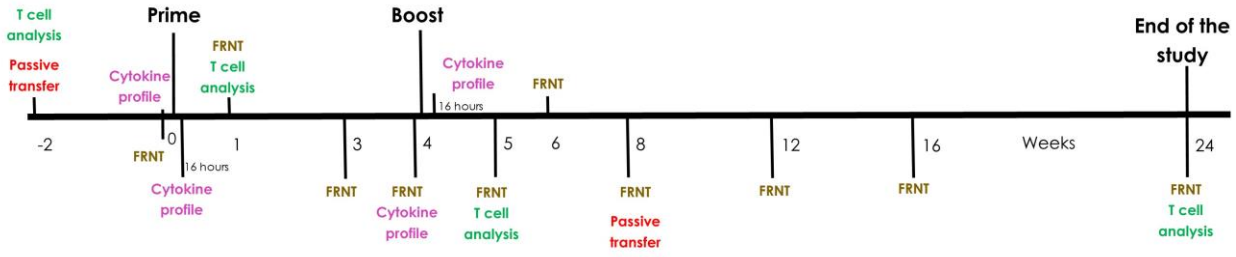

e

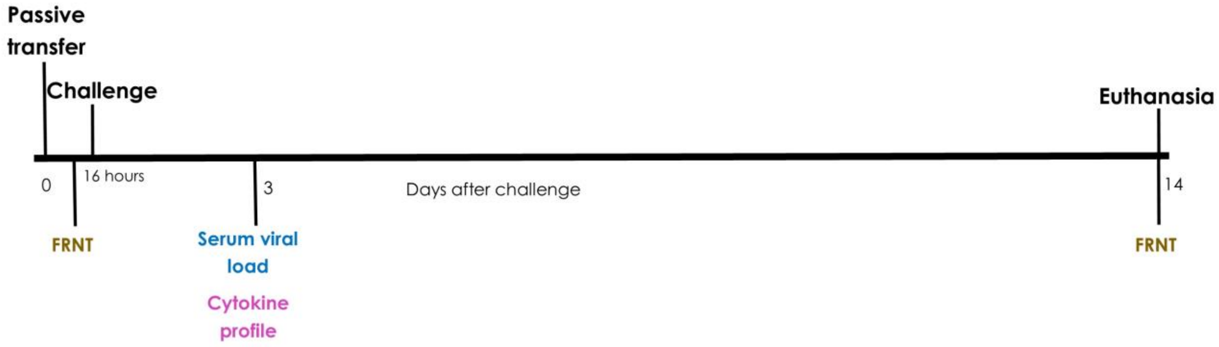

Supplementary figure 1. Animal treatment schedule and sample collection. (a) In mouse vaccination studies, mice received two immunizations via IM injection 3 weeks apart (day 0, 21), except for 2.5 prM-E/NS1 and YF-17D vaccine groups which received a single immunization on day 0. All mice were challenged in week 5 (day 35) and sera were obtained after 3, 4, 5, 6, and 7 (end of the study) weeks. **(b)** For passive transfer studies, mice received 200 µL of serum via IP injection and were bled and challenged after 12 h. Additional serum samples were taken on days 6 and 14 after challenge (end of the study). **(c)** For adoptive transfer of splenocytes, mice received 1x10^7 splenocytes from vaccinated or placebo mice via retro-orbital injection. Recipient mice were bled and challenged after 16 h. Mice were bled on days 3 and 14 (end of the study). **(d)** Macaques received two doses of mRNA-LNP vaccines 4 weeks apart (days 0, 28) via IM injection or a single dose of YFV-17D vaccine on day 0. Serum samples were obtained in weeks -2 and 0 (before study start), 16 h after each vaccination (d1 or d29) and in weeks 1, 3, 4, 5, 6, 8, 12, 16, and 24 (end of the study). PBMCs were obtained in weeks -2, 1, 5, and 24. **(e)** For passive transfer of sera from macaques into A129 mice, NHP serum samples from week -2 (naïve) or week 8 (vaccinated) were used. Mice received 200 µL of serum via IP injection and were bled and challenged after 16 h. Additional blood samples were taken from infected mice on days 3 and 14 (end of the study).

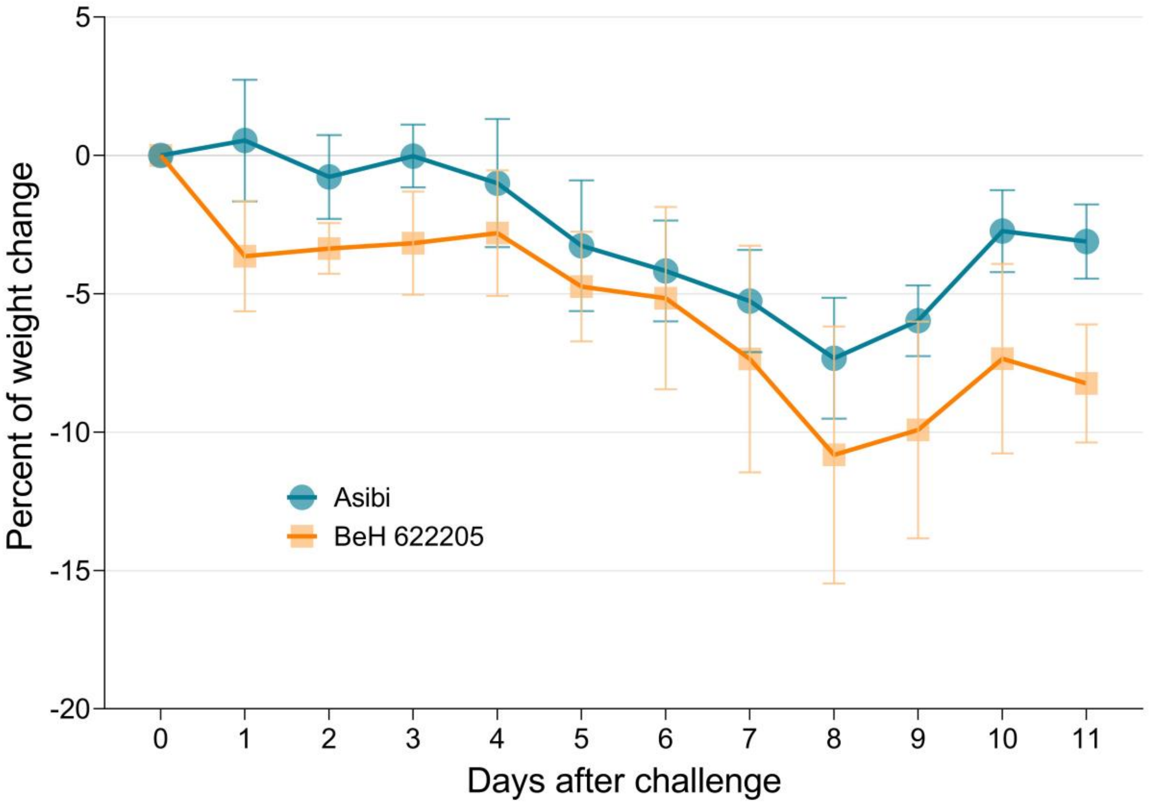

Supplementary figure 2. Pathogenicity of wtYFV strains in adult A129 mice. Eight to eleven weeks old A129 mice were infected via IM injection with $1x10^4$ PFU of YFV Asibi (*n=3*) or BeH 622205 (*n=5*) strains. The graph shows the percentage of the body weight change (± SD) of the animals over a period of 11 days post-challenge.
