# Supplementary material for: Immunogenicity and protective activity of mRNA vaccine candidates against yellow fever virus in animal models
Source: NPJ Vaccines. 2023 Mar 4;8:31. doi: 10.1038/s41541-023-00629-7 (PMC9984760; doi:10.1038/s41541-023-00629-7)
Supplement: Supplementary file 2 — Reporting Summary [file 41541_2023_629_MOESM2_ESM.pdf]

nature portfolio

Corresponding author(s): Lex G. Medina-Magües, Dr Jorge E. Osorio

Last updated by author(s): Feb 1, 2023

Reporting Summary

Nature Portfolio wishes to improve the reproducibility of the work that we publish. This form provides structure for consistency and transparency in reporting. For further information on Nature Portfolio policies, see our Editorial Policies and the Editorial Policy Checklist.

Statistics

For all statistical analyses, confirm that the following items are present in the figure legend, table legend, main text, or Methods section.

n/a	Confirmed	
☐	☒	The exact sample size (*n*) for each experimental group/condition, given as a discrete number and unit of measurement
☒	☐	A statement on whether measurements were taken from distinct samples or whether the same sample was measured repeatedly
☐	☒	The statistical test(s) used AND whether they are one- or two-sided<br>*Only common tests should be described solely by name; describe more complex techniques in the Methods section.*
☐	☒	A description of all covariates tested
☐	☒	A description of any assumptions or corrections, such as tests of normality and adjustment for multiple comparisons
☐	☒	A full description of the statistical parameters including central tendency (e.g. means) or other basic estimates (e.g. regression coefficient) AND variation (e.g. standard deviation) or associated estimates of uncertainty (e.g. confidence intervals)
☐	☒	For null hypothesis testing, the test statistic (e.g. *F*, *t*, *r*) with confidence intervals, effect sizes, degrees of freedom and *P* value noted<br>*Give P values as exact values whenever suitable.*
☒	☐	For Bayesian analysis, information on the choice of priors and Markov chain Monte Carlo settings
☒	☐	For hierarchical and complex designs, identification of the appropriate level for tests and full reporting of outcomes
☒	☐	Estimates of effect sizes (e.g. Cohen's *d*, Pearson's *r*), indicating how they were calculated

Our web collection on statistics for biologists contains articles on many of the points above.

Software and code

Policy information about availability of computer code

Data collection	Viridot software v1.0, CTL ImmunoSpot® Software v7.0.9.5, xPONENT® software v4.2, iCycler iQ Software v3.1, Gen5 software v3.04.17.
Data analysis	GraphPad Prism v9.3.0 Software

For manuscripts utilizing custom algorithms or software that are central to the research but not yet described in published literature, software must be made available to editors and reviewers. We strongly encourage code deposition in a community repository (e.g. GitHub). See the Nature Portfolio guidelines for submitting code & software for further information.

Data

Policy information about availability of data

All manuscripts must include a data availability statement. This statement should provide the following information, where applicable:

- Accession codes, unique identifiers, or web links for publicly available datasets
- A description of any restrictions on data availability
- For clinical datasets or third party data, please ensure that the statement adheres to our policy

Data supporting the findings of this study are available in this paper, Supplementary Material, or are available from the corresponding authors upon request.

Human research participants

Policy information about studies involving human research participants and Sex and Gender in Research.

Reporting on sex and gender	*Use the terms sex (biological attribute) and gender (shaped by social and cultural circumstances) carefully in order to avoid confusing both terms. Indicate if findings apply to only one sex or gender; describe whether sex and gender were considered in study design whether sex and/or gender was determined based on self-reporting or assigned and methods used. Provide in the source data disaggregated sex and gender data where this information has been collected, and consent has been obtained for sharing of individual-level data; provide overall numbers in this Reporting Summary. Please state if this information has not been collected. Report sex- and gender-based analyses where performed, justify reasons for lack of sex- and gender-based analysis.*
Population characteristics	*Describe the covariate-relevant population characteristics of the human research participants (e.g. age, genotypic information, past and current diagnosis and treatment categories). If you filled out the behavioural & social sciences study design questions and have nothing to add here, write "See above."*
Recruitment	*Describe how participants were recruited. Outline any potential self-selection bias or other biases that may be present and how these are likely to impact results.*
Ethics oversight	*Identify the organization(s) that approved the study protocol.*

Note that full information on the approval of the study protocol must also be provided in the manuscript.

Field-specific reporting

Please select the one below that is the best fit for your research. If you are not sure, read the appropriate sections before making your selection.

☒ Life sciences ☐ Behavioural & social sciences ☐ Ecological, evolutionary & environmental sciences

For a reference copy of the document with all sections, see nature.com/documents/nr-reporting-summary-flat.pdf

Life sciences study design

All studies must disclose on these points even when the disclosure is negative.

Sample size	We have minimized the number of animals per group and the number of experimental groups while still permitting scientifically valid conclusions. The number of animals chosen is required to compare antibody and viral titers accurately. Power analysis was conducted to determine the necessary optimal number of animals to maintain the desired power of 80% for the study (α=0.05). The total includes minimal numbers of additional animals to account for a repeat study if necessary due to unforeseen circumstances. Furthermore, such numbers are not uncommon among animal vaccine studies within our lab and externally. The sample size used for the different studies and groups were described in the result and material sections of the manuscript.
Data exclusions	No data were excluded, except the analyte G-CSF (CSF-3) because of the low number of beads detected (<32).
Replication	The YFV challenge was used and reported in the manuscript three times and showed a high reproducibility. All animal vaccinations and passive transfers of serum were also highly reproducible after a total of five studies described in the manuscript.
Randomization	The manuscript described that the mice were randomly allocated to groups. All mice were pooled together in a large cage per each sex and were randomly allocated to the different vaccine treatment groups. As we tried to achieve equal representation of female and male mice per each group, two cages (one per each sex) per treatment group was used. The use of female and male treatment cages prevented the breeding of the mice. For the primate study, the animals were blindingly chosen from a list with alphanumeric ID tags to allocate them for the vaccine treatment groups. Primates were kept in individual cages and in different rooms.
Blinding	All animals were given numeric ID tags that didn't represent any specific treatment group.

Reporting for specific materials, systems and methods

We require information from authors about some types of materials, experimental systems and methods used in many studies. Here, indicate whether each material, system or method listed is relevant to your study. If you are not sure if a list item applies to your research, read the appropriate section before selecting a response.

Materials & experimental systems

n/a	Involved in the study	
☐	☒	Antibodies
☐	☒	Eukaryotic cell lines
☒	☐	Palaeontology and archaeology
☐	☒	Animals and other organisms
☒	☐	Clinical data
☒	☐	Dual use research of concern

Methods

n/a	Involved in the study	
☒	☐	ChIP-seq
☒	☐	Flow cytometry
☒	☐	MRI-based neuroimaging

Antibodies

Antibodies used	YFV hyperimmune mouse ascitic fluid (provided by the World Reference Center for Emerging Viruses and Arboviruses at the University of Texas Medical Branch), horseradish peroxidase-conjugated goat anti-mouse IgG (H + L) (Invitrogen, Cat. No. 31430), horseradish peroxidase-conjugated goat anti-human IgG (H + L) (Promega, Cat. No. W4031)
Validation	Antibodies were validated and titrated for specificity prior to use in each assay.

Eukaryotic cell lines

Policy information about cell lines and Sex and Gender in Research

Cell line source(s)	HeLa (ATCC: CCL-2) derived from a female Homo sapiens and Vero (ATCC: CCL-81) cells derived from an adult Cercopithecus aethiops.
Authentication	HeLa (ATCC: CCL-2) and Vero (ATCC: CCL-81) cells were purchased and authenticated by ATCC.
Mycoplasma contamination	Both cell lines came mycoplasma free from the manufacturer and were routinely tested for mycoplasma after being expanded and used in the lab.
Commonly misidentified lines (See ICLAC register)	*Name any commonly misidentified cell lines used in the study and provide a rationale for their use.*

Animals and other research organisms

Policy information about studies involving animals; ARRIVE guidelines recommended for reporting animal research, and Sex and Gender in Research

Laboratory animals	A129 mice of 129/SvEv genetic background were bred in pathogen-free animal facilities at UW-Madison School of Veterinary Medicine. Indian rhesus macaques (Macaca mulatta), 2-3 years of age, weighing 3-6kg were handled by qualified personnel from the Wisconsin National Primate Research Center veterinary staff.
Wild animals	Study did not involve wild animals.
Reporting on sex	All findings apply to both sexes combined. For the animals studies using mice, the authors reported the term "mixed-sex" to explain the use of 50% of female and 50% male mice per treatment group. For the primate study, the authors reported that each treatment group consisted of 3 female and 3 male Indian rhesus macaques.
Field-collected samples	Study did not involve sample collection from the field.
Ethics oversight	All animal studies followed the guidelines described in the National Research Council's Guide for the Care and Use of Laboratory Animals, the Animal Welfare Act and the Guide for the Care and Use of Laboratory Animals, and the recommendations of the Weatherall report. All animal research was conducted under the authority of the UW-Madison School of Veterinary Medicine and supervised by the UW-Madison Research Animal Resources and Compliance. The protocol (# G005519-R01-A01) was approved by the UW-Madison Institutional Animal Care and Use Committee. Rhesus macaques were handled by qualified personnel from the Wisconsin National Primate Research Center veterinary staff.

Note that full information on the approval of the study protocol must also be provided in the manuscript.
